# Associations of oxygenated hemoglobin with disease burden and prognosis in stable COPD: Results from COSYCONET

**DOI:** 10.1038/s41598-020-67197-x

**Published:** 2020-06-29

**Authors:** F. C. Trudzinski, R. A. Jörres, P. Alter, K. Kahnert, B. Waschki, C. Herr, C. Kellerer, A. Omlor, C. F. Vogelmeier, S. Fähndrich, H. Watz, T. Welte, B. Jany, S. Söhler, F. Biertz, F. Herth, H.-U. Kauczor, R. Bals, the COSYCONET consortium, Stefan Andreas, Jürgen Behr, Burkhard Bewig, Roland Buhl, Ralf Ewert, Beate Stubbe, Joachim H. Ficker, Manfred Gogol, Christian Grohé, Rainer Hauck, Matthias Held, Markus Henke, Gerd Höffken, Hugo A. Katus, Anne-Marie Kirsten, Rembert Koczulla, Klaus Kenn, Juliane Kronsbein, Kropf-Sanchen, Christoph Lange, Peter Zabel, Michael Pfeifer, Winfried J. Randerath, Werner Seeger, Michael Studnicka, Christian Taube, Helmut Teschler, Hartmut Timmermann, J. Christian Virchow, Wagner, Hubert Wirtz

**Affiliations:** 1 Department of Internal Medicine V - Pulmonology, Allergology, Critical Care Care Medicine, Saarland University Hospital, Homburg, Germany; 2 Institute and Outpatient Clinic for Occupational, Social and Environmental Medicine, Ludwig Maximilians University (LMU), Comprehensive Pneumology Center Munich (CPC-M), Member of the German Center for Lung Research (DZL), Munich, Germany; 3 Department of Medicine, Pulmonary and Critical Care Medicine, Philipps University of Marburg (UMR), Member of the German Center for Lung Research (DZL), Marburg, Germany; 4 Department of Internal Medicine V, University Hospital, LMU Munich, Comprehensive Pneumology Center, Member of the German Center for Lung Research (DZL), Munich, Germany; 5 Department of General and Interventional Cardiology, University Heart Center Hamburg, Hamburg, Germany; 6 Pulmonary Research Institute at LungenClinic Grosshansdorf, Airway Research Center North (ARCN), Member of the German Center for Lung Research (DZL), Grosshansdorf, Germany; 7 TUM School of Medicine, Institute of General Practice and Health Services Research, Technical University of Munich, Orleansstraße 47, 81667 Munich, Germany; 8 Department of Pneumology, University Hospital Freiburg, Freiburg, Germany; 9 Clinic for Pneumology, Hannover Medical School, Biomedical Research in Endstage and Obstructive Lung Disease Hannover (BREATH), Member of the German Center for Lung Research, Hannover, Germany; 10 Department of Internal Medicine, Medical Mission Hospital, Academic Teaching Hospital, Julius Maximilian University of Würzburg, Würzburg, Germany; 11 Institute for Biostatistics, Hannover Medical School, Hannover, Germany; 12 Department of Pneumology and Critical Care Medicine, Thoraxklinik University of Heidelberg, Translational Lung Research Center Heidelberg (TLRC-H), Member of the German Center for Lung Research (DZL), Heidelberg, Germany; 13 Department of Diagnostic and Interventional Radiology, Heidelberg University Hospital, Translational Lung Research Center Heidelberg (TLRC-H) member of the German Center of Lung Research, Heidelberg, Germany; 14 Lungenfachklinik, Immenhausen, Germany; 15 Universitätsklinikum Schleswig Holstein, Campus Kiel, Germany; 16 Universitätsmedizin der Johannes-Gutenberg-Universität Mainz, Mainz, Germany; 17 Universitätsmedizin Greifswald, Greifswald, Germany; 18 Klinikum Nürnberg, Paracelsus Medizinische Privatuniversität Nürnberg, Nürnberg, Germany; 19 Institut für Gerontologie, Universität Heidelberg, Heidelberg, Germany; 20 Ev. Lungenklinik Berlin, Berlin, Germany; 21 Kliniken Südostbayern AG, Kreisklinik Bad Reichenhall, Bad Reichenhall, Germany; 22 Asklepios Fachkliniken München-Gauting, Munich, Germany; 23 Fachkrankenhaus Coswig GmbH, Coswig, Germany; 24 Department of Cardiology, Angiology and Pneumology, University of Heidelberg, Heidelberg, Germany; 25 Schön Klinik Berchtesgadener Land, Schönau am Königssee, Germany; 26 Berufsgenossenschaftliches Universitätsklinikum Bergmannsheil, Bochum, Germany; 27 Universitätsklinikum Ulm, Ulm, Germany; 28 Forschungszentrum Borstel, Borstel, Germany; 29 Klinik Donaustauf, Donaustauf, Germany; 30 Wissenschaftliches Institut Bethanien e. V., Solingen, Germany; 31 Justus-Liebig-Universität Gießen, Gießen, Germany; 32 Uniklinikum Salzburg, Salzburg, Salzburg, Austria; 33 Ruhrlandklinik gGmbH Essen, Essen, Germany; 34 Hamburger Institut für Therapieforschung GmbH, Hamburg, Germany; 35 Universitätsklinikum Rostock, Rostock, Germany; 36 Klinik Löwenstein gGmbH, Löwenstein, Germany; 37 Universitätsklinikum Leipzig, Leipzig, Germany

**Keywords:** Biomarkers, Medical research, Risk factors, Signs and symptoms

## Abstract

We studied whether in patients with stable COPD blood gases (BG), especially oxygenated hemoglobin (OxyHem) as a novel biomarker confer information on disease burden and prognosis and how this adds to the information provided by the comorbidity pattern and systemic inflammation. Data from 2137 patients (GOLD grades 1–4) of the baseline dataset of the COSYCONET COPD cohort were used. The associations with dyspnea, exacerbation history, BODE-Index (cut-off ≤2) and all-cause mortality over 3 years of follow-up were determined by logistic and Cox regression analyses, with sex, age, BMI and pack years as covariates. Predictive values were evaluated by ROC curves. Capillary blood gases included SaO_2_, PaO_2_, PaCO_2_, pH, BE and the concentration of OxyHem [haemoglobin (Hb) x fractional SaO_2_, g/dL] as a simple-to-measure correlate of oxygen content. Inflammatory markers were WBC, CRP, IL-6 and -8, TNF-alpha and fibrinogen, and comorbidities comprised a broad panel including cardiac and metabolic disorders. Among BG, OxyHem was associated with dyspnoea, exacerbation history, BODE-Index and mortality. Among inflammatory markers and comorbidities, only WBC and heart failure were consistently related to all outcomes. ROC analyses indicated that OxyHem provided information of a magnitude comparable to that of WBC, with optimal cut-off values of 12.5 g/dL and 8000/µL, respectively. Regarding mortality, OxyHem also carried independent, additional information, showing a hazard ratio of 2.77 (95% CI: 1.85–4.15, p < 0.0001) for values <12.5 g/dL. For comparison, the hazard ratio for WBC > 8000/µL was 2.33 (95% CI: 1.60–3.39, p < 0.0001). In stable COPD, the concentration of oxygenated hemoglobin provided additional information on disease state, especially mortality risk. OxyHem can be calculated from hemoglobin concentration and oxygen saturation without the need for the measurement of PaO_2_. It thus appears well suited for clinical use with minimal equipment, especially for GPs.

## Introduction

Chronic obstructive pulmonary disease (COPD) is a progressive, debilitating condition and estimated to become the third-leading cause of death worldwide in 2020^1^. Major contributors to disease status are recurrent exacerbations, driven by respiratory infections, and multiple functional impairments and comorbidities^2–4^. These alterations are reflected in blood gases (BG) values and the degree of renal compensation of acid-base imbalance. Various indices characterizing this imbalance, such as base excess (BE), are routinely used as indicators of respiratory impairment during acute exacerbations^5,6^.

Much less is known on the usefulness of BG in stable COPD, and a detailed analysis of their predictive value seems worthwhile. A recent study showed that especially oxygen content (CaO_2_) conferred information on the exacerbation risk in stable COPD^7^. Due to the low amount of solved oxygen, CaO_2_ is very closely proportional to the concentration of oxygenated hemoglobin, which can be calculated without need for the assessment of PaO_2_. Beyond acute impairments, BG might reflect the long-term burden of both persistent functional impairments and frequent exacerbations with incomplete recovery. As putative integrative markers they could ameliorate some of the difficulties encountered in clinical practice when retrospectively assessing the history of exacerbations in an individual patient^8,9^. COPD is also associated with systemic inflammation, which is linked to exacerbation risk^10^ and probably to distortions of BG. It therefore seems reasonable to compare the impact of BG with that of systemic inflammation and comorbidities.

We thus investigated the role of BG for disease burden and prognosis in patients with stable COPD, either alone or combined with comorbidities and markers of inflammation, putting particular emphasis on the concentration of oxygenated hemoglobin (OxyHem) as a potentially useful, novel marker. The outcome measures were symptoms and exacerbation history according to GOLD, the BODE-Index (Body mass index, airflow Obstruction, Dyspnea, Exercise capacity), and mortality^11^. All data used came from the prospective COPD cohort COSYCONET (Systemic Consequences - Comorbidities Network)^12^.

## Methods

### Study population

COSYCONET enrolled 2741 patients with COPD (age ≥ 40 years) throughout Germany between 2010 and 2013^12^. The study was conducted in accordance with the amended Declaration of Helsinki. All assessments were approved by the central [Marburg (Ethikkommission FB Medizin Marburg)] and local [Bad Reichenhall (Ethikkommission bayerische Landesärztekammer); Berlin (Ethikkommission Ärztekammer Berlin); Bochum (Ethikkommission Medizinische Fakultät der RUB); Borstel (Ethikkommission Universität Lübeck); Coswig (Ethikkommission TU Dresden); Donaustauf (Ethikkommission Universitätsklinikum Regensburg); Essen (Ethikkommission Medizinische Fakultät Duisburg-Essen); Gießen (Ethikkommission Fachbereich Medizin); Greifswald (Ethikkommission Universitätsmedizin Greifswald); Großhansdorf (Ethikkommission Ärztekammer Schleswig-Holstein); Hamburg (Ethikkommission Ärztekammer Hamburg); MHH Hannover/Coppenbrügge (MHH Ethikkommission); Heidelberg Thorax/Uniklinik (Ethikkommission Universität Heidelberg); Homburg (Ethikkommission Saarbrücken); Immenhausen (Ethikkommission Landesärztekammer Hessen); Kiel (Ethikkommission Christian-Albrechts-Universität zu Kiel); Leipzig (Ethikkommission Universität Leipzig); Löwenstein (Ethikkommission Landesärztekammer Baden-Württemberg); Mainz (Ethikkommission Landesärztekammer Rheinland-Pfalz); München LMU/Gauting (Ethikkommission Klinikum Universität München); Nürnberg (Ethikkommission Friedrich-Alexander-Universität Erlangen Nürnberg); Rostock (Ethikkommission Universität Rostock); Berchtesgadener Land (Ethikkommission Land Salzburg); Schmallenberg (Ethikkommission Ärztekammer Westfalen-Lippe); Solingen (Ethikkommission Universität Witten-Herdecke); Ulm (Ethikkommission Universität Ulm); Würzburg(Ethikkommission Universität Würzburg] ethical committees and written informed consent was obtained from all patients. Participants were required to be in a stable clinical condition without exacerbation within four weeks preceding the study visit^12^. The present analysis took data from the baseline visit of patients with GOLD grades 1–4^3,4^ (N = 2387). Among these, data of 2137 patients with valid BG and prospective mortality data were analyzed.

### Pulmonary function tests, exacerbations, BODE-Index

Following the COSYCONET study protocol^12^, spirometry and body plethysmography were performed in line with recommendations^13–17^ after bronchodilator inhalation (400 µg salbutamol and 80 µg ipratropium bromide). Global Lung Function Initiative (GLI) and European Community for Steel and Coal (ECSC) reference values were used^14,18^ but lung function served only for the description of the population and was not topic of the analysis.

GOLD groups ABCD were formed on the basis of the Modified Medical Research Council dyspnea scale (mMRC)^19^ and exacerbation history^20^. Patients, who reported ≥2 exacerbations without hospital admission or ≥1 exacerbation leading to hospital admission within the year before the visit, were categorized as groups C or D, depending on symptoms. For symptoms, the mMRC cutoff value of 2 was used^20^. Based on the ABCD groups, a binary exacerbation history variable was defined combining groups C/D (high risk) versus A/B (low risk), and a binary symptom variable combining groups B/D (high symptom) versus A/C (low symptoms), similarly as done previously^2^. The BODE-Index was determined following the recommendations^11^, the 6-min walk distance (6-MWD) as described in the American Thoracic Society (ATS) guidelines^21^. A binary BODE-Index was defined by dichotomizing the 10-point scale at ≤2 or >2. This cut-off value was chosen, as it resulted in balanced groups and turned out to be most informative when comparing different cut-off values. We included the BODE-Index under the hypothesis that this score, which is known to be predictive for mortality, might yield results intermediate between symptoms/exacerbations and mortality risk und thus help to understand the findings.

### Blood gas analysis

The values of PaO_2_, PaCO_2_, pH and SaO_2_ were obtained from arterialized capillary blood from the earlobe. This method can be used instead of blood obtained by arterial puncture based on results described by Langlands *et al*. in 1965^22^

Adequate sampling, as well as calibration and quality control of blood gas analysers, were ensured by standardized operating procedures (SOP). The earlobe was pre-warmed with application of a vasoactive cream to assure a sufficient vasodilatation. Blood gas analyzers were those available in the study centers (e-Appendix 3).

Base excess (BE) values were taken from the integrated algorithms. CaO_2_ was calculated as 1.34 x Hb x fractional SaO_2_ + 0.0031 x PaO_2_, with Hb indicating hemoglobin concentration^23^. As a novel marker, the concentration of oxygenated hemoglobin (termed “OxyHem”, g/dL) was calculated as Hb x fractional SaO_2_. This measure is practically equivalent to CaO_2_, as the variation of CaO_2_ from solved oxygen is less than 1% of total CaO_2_ but it is attractive when searching for most simple markers among BG parameters.

### Inflammatory biomarkers

WBC count and C-reactive protein (CRP) were determined in the laboratories of the study centers using quality-controlled procedures. Concentrations of fibrinogen, interleukin-6 (IL-6), −8 (IL-8), and tumor necrosis factor alpha (TNF-α) were determined in the central biobank following the manufacturers’ instructions (for details see supplement).

### Comorbidities

In COSYCONET, a broad panel of comorbidities was assessed by structured interviews based on patients’ reports of physician-based diagnoses^12^. Moreover, the presence of disease-specific medication was taken into account^24^. The comorbidities included in the present analysis comprised diabetes, hyperlipidemia, hyperuricemia, gastrointestinal disorders, hypertension, coronary artery disease, heart failure, osteoporosis, psychiatric disorders, sleep apnea, and asthma.

### Mortality

All-cause mortality was assessed over 3 years of follow-up. After baseline, patients were invited for each follow-up visit via telephone and letters. If a patient missed a follow-up visit without formally withdrawing from the study, research assistants ascertained the survival status by contacting partners, relatives, primary care practitioners and hospitals as described previously^25^.

### Data analysis

Data in the tables are presented as numbers and percentages, or mean values, minimum, maximum and standard deviations (SD). The associations of blood gases, inflammatory markers and comorbidities with symptom burden, exacerbation history and binary BODE-Index were analysed via logistic regression analyses, always keeping sex, age, BMI and pack years as covariates. In addition, for the non-binary BODE-Index, a multiple linear regression analysis was performed to estimate the magnitude of effects. An analogous Cox proportional hazard regression analysis served for the identification of prognostic factors for mortality. These analyses were performed separately with the three sets of measures of BG, or inflammation, or comorbidities. Among BG measures, OxyHem was included instead of CaO_2_. The predictive value of BG measures, either alone or in combination with comorbidities or inflammatory markers, was further evaluated by ROC analyses, using the probabilities obtained in the logistic regression analyses with each of the sets of predictors. If single predictors were analyzed, the Youden-Index was used to derive cut-off values.

The final Cox regression analysis for mortality risk comprised only the covariates and selected, significant predictors from BG, comorbidities and inflammatory markers. It was repeated while including the BODE-Index and/or intake of oral or inhaled corticosteroids and/or Hb concentration or SaO_2_ as additional predictors, in order to reveal whether the predictors identified as relevant were still significant. All analyses were performed with SPSS version 25 (IBM Corp., Armonk, NY, USA); p values < 0.05 were considered as statistically significant.

## Results

### Study cohort

The characteristics of the 2137 participants are given in Table 1, including their distribution over GOLD grades 1–4 and groups A-D, lung function data, BG measures and inflammatory markers. Table 2 shows the prevalence of comorbidities. LTOT was present in 207 (9.7%) patients and there were 541 (25.3%) current smokers.

**Table 1:** Baseline characteristics of the study cohort (n = 2137).

Variable	Mean	SD	Minimum	Maximum
**Anthropometric characteristics**				
**Sex (%)**				
male	1301 (60.9)	—	—	—
female	836 (39.1)	—	.	—
Age (y)	64.9	8.42	40.0	90.0
BMI (kg/m²)	26.7	5.24	12.9	56.0
Pack years	49.1	35.9	0.00	270.0
**Pulmonary function tests**				
FEV_1**%**_pred (GLI)	53.1	18.5	13.0	121.0
FVC %pred (GLI)	78.8	19.0	21.6	144.1
FRC %pred (ECSC)	149.9	35.7	62.1	349
RV %pred (ECSC)	173.1	52.3	33.5	482
**GOLD stages and BODE Index**				
GOLD * (%)	.	—	—	—
1	200 (9.4)	—	—	—
2	921 (43.1)	—	—	—
3	805 (37.7)	—	—	—
4	211 (9.9)			
GOLD ** (%)	.	—	—	—
A	846 (39.6)	—	—	—
B	528 (24.7)	—	—	—
C	285 (13.3)	—	—	—
D	478 (22.4)			
BODE-Index	2.47	1.99	0	9
**Blood gases**				
PaO_2_ (mmHg)	66.7	8.55	39.9	107.0
PaCO_2_ (mmHg)	37.9	4.66	23.2	60.0
pH	7.43	0.03	7.35	7.60
BE (mmol/L)	1.17	2.31	−9.10	14.0
CaO_2_ (mL/dL)	18.7	1.73	11.4	26.9
OxyHem (g/dL)	13.8	1.29	8.31	19.4
**Inflammatory markers**				
Hb (g/dL)	14.7	1.36	8.80	21.7
WBC (1000/µL)	8.01	2.39	2.60	41.9
CRP (mg/dL)	1.03	2.89	0.01	74.8
Fibrinogen (g/L)	2.66	1.27	0.00	12.5
IL-6 (pg/mL)	13.0	93.5	0.20	3454
IL-8 (pg/mL)	13.8	86.3	0.05	3742
TNF-α (pg/mL)	11.0	18.9	0.07	418

The table shows mean values, standard deviations (SD), minimum and maximum values, or absolute numbers in case of sex and GOLD stages and GOLD groups, *based on GLI predicted values, **based on mMRC; FRC functional residual capacity determined in body plethysmography, RV residual volume determined in body plethysmography.

**Table 2:** Prevalence of comorbidities in the study cohort (n = 2137).

Comorbidity	Number (%)
Hypertension	1204 (56.3)
Gastrointestinal disorders	985 (46.1)
Hyperlipidemia	915 (42.8)
Psychiatric disorders	516 (24.1)
Asthma	406 (19.0)
Hyperuricemia	377 (17.6)
Coronary artery disease	363 (17.0)
Osteoporosis	340 (15.9)
Diabetes	276 (12.9)
Sleep apnea	228 (10.7)
Heart failure	110 (5.1)

The table shows absolute numbers and percentages. Comorbidities were assessed by structured interviews based on patients’ reports of physician-based diagnoses. For all comorbidities except cardiac failure and sleep apnea, the presence of disease-specific medication was additionally taken into account.

### Symptoms, exacerbation history and BODE-Index

**Figure 1:**
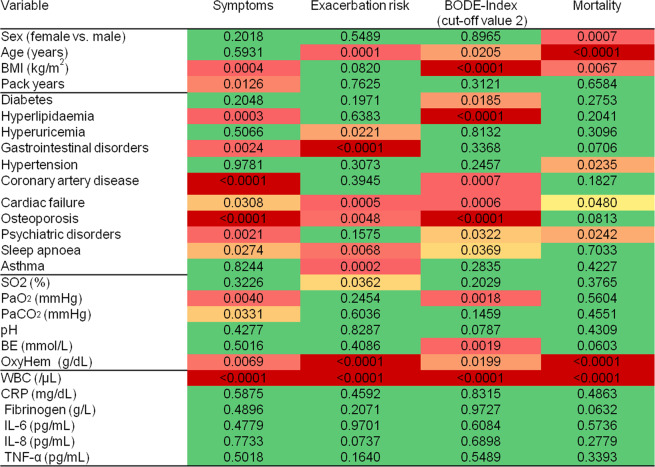
Heatmap of associations between comorbidities, blood gases and systemic inflammation with symptom burden (GOLD mMRC)), exacerbation history (GOLD), BODE Index (cut-off value 2) and mortality analyzed by logistic regression analyses. The figure shows p values as derived from multiple regression analyses. The p values for the anthropometric characteristics refer to those obtained from the blood gas analyses. Colors indicate the strength of the associations (from green, p ≥ 0.05, to dark red, strongly significant, p ≤ 0.0001).

The associations of comorbidities, BG and inflammatory markers with symptom burden, exacerbation historyand the binary BODE-Index are presented as a heatmap in Fig. 1. It shows the respective p values, with colors indicating the strength of associations (see legend). Regarding comorbidities, symptoms were related to cardiovascular comorbidities, osteoporosis, gastrointestinal and psychiatric disorders. Similar findings were obtained for the binary BODE-Index. Symptoms and BODE-Index were also linked to hyperlipidemia, whereby its effect was beneficial. Exacerbation history was associated with hyperuricemia, gastrointestinal disorders, heart failure, osteoporosis, sleep apnea, and concomitant asthma.

**Figure 2:**
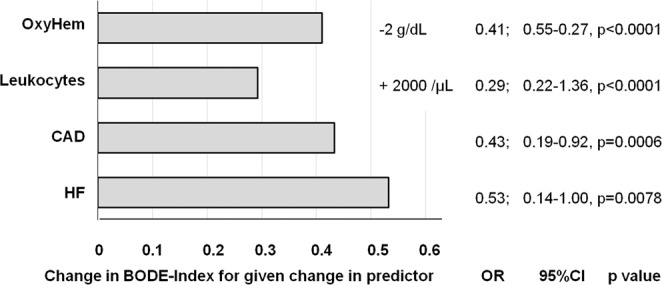
The figure shows the absolute change of BODE-Index as derived from multiple linear regression analysis adjusting for sex, age, BMI and pack years for defined changes in four selected predictors. The change is given for a change in OxyHem by −2 g/dL, in WBC count by +2000/µL, or the presence of coronary artery disease (CAD) or heart failure (HF). Additionally, the numerical values of the changes in the BODE-Index, their 95% confidence intervals and the corresponding p values are shown.

Among BG, OxyHem was the strongest predictor and associated with all three outcome measures mentioned. PaO_2_ and BE were additionally associated with the binary BODE-Index. Among inflammatory markers, only WBC counts were consistently linked to the three outcome measures. The absolute changes in BODE-Index predicted by a reduction in OxyHem by 2 g/dL, or an increase in WBC count by 2000/µL were similar to the effects of coronary artery disease (CAD) or heart failure (HF) (Fig. 2**)**.

### Mortality

Overall, 130 patients (6.1%) died during the follow-up. The results of the Cox regression analyses are included in Fig. 1. We identified arterial hypertension (HR 1.60, 95% CI: 1.07–2.40, p = 0.0235), heart failure (HR 1.83, 1.01–3.34, p = 0.0480) and mental disorders (HR 1.63, 1.07–2.48, p = 0.0242) as prognostic factors for mortality, independently from each other and sex, age, BMI and pack years **(**e-Table 1**)**. OxyHem (HR 0.747 for +1 g/dl, 0.69–0.91, p = 0.0002) turned out as prognostic factor among BG **(**e-Table 2**)**. WBC count (HR 1.10 for +1000/µL, 1.06–1.14, p < 0.0001) **(**e-Table 3**)** among the inflammatory markers.

### Receiver operating characteristics

ROC analyses underlined that for symptoms, exacerbation history and binary BODE-Index, BG provided additional information compared to comorbidities or inflammatory markers. For symptoms, area under the curve (AUC) was highest for the combination of BG with comorbidities (0.679, 0.618–0.670, p < 0.0001). For exacerbation history, the combination of comorbidities and inflammatory markers was most predictive (0.673, 0.646–0.699, p < 0.0001), closely followed by BG combined with comorbidities (0.660, 0.633–0.678, p < 0.0001). The binary BODE-Index showed the highest AUC for the combination of BG with comorbidities (0.711, 0.686–0.736, p < 0.0001). For ROC curves, the respective AUC and 95% Wilson confidence intervals see the supplementary files (e-Fig. 1, e-Table 4).

### Simplified blood gas parameter OxyHem in comparison to WBC count

**Figure 3:**
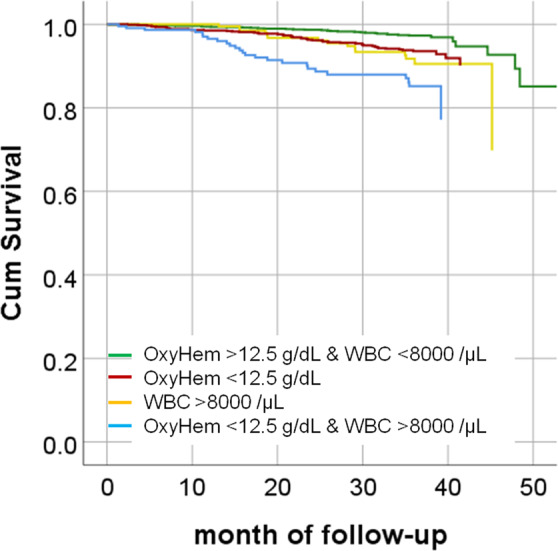
Cox proportional hazards cumulative survival curves stratified for either OxyHem <12.5 g/dL, or WBC count >8000/µL. The corresponding hazard ratios for the binary OxyHem were 2.77 (95% CI: 1.85–4.15, p < 0.0001), and for the binary WBC count 2.33 (95% CI: 1.60–3.39, p < 0.0001 s), respectively. We additionally show the combined value in the sense, that either both measures were on the side of elevated risk, or both not, in order to demonstrate their combined value.

Regarding the binary BODE-Index and taking the Youden-Index as criterion, the optimal cut-off value of OxyHem was 12.5 g/dL, while that for WBC was 8000/µL. These two measures were compared regarding their predictive value for mortality via Cox regression analysis, omitting all other BG values, inflammatory markers, and comorbidities. In the presence or absence of the covariates sex, age, BMI and pack years, OxyHem and WBC count were both relevant outcome variables, without significant interaction terms. This was true for both the continuous variables and their binary reductions based on OxyHem <12.5 g/dL and WBC > 8000/µL. Taking these cut-off values and the covariates mentioned, the binary OxyHem showed a HR for mortality of 2.77 (95% CI: 1.85–4.15, p < 0.0001) and the binary WBC of 2.33 (1.60–3.39, p < 0.0001) (Table 3**)**. Survival curves for binary OxyHem, binary WBC and their combination are shown in Fig. 3.

**Table 3:** Cox regression analysis for mortality risk (n = 2137).

Predictor	B	SE	HR	95%CI for HR		P value
				Lower	Upper	
Sex (f vs m)	−0.63	0.22	0.53	0.34	0.83	0.005
Age (y)	0.07	0.01	1.07	1.05	1.10	**<0.0001**
BMI (kg/m^2^)	−0.06	0.02	0.94	0.90	0.98	0.006
Packyears	0.00	0.00	1.00	0.99	1.00	0.599
OxyHem <12.5 g/dL	1.02	0.21	2.77	1.85	4.15	**<0.0001**
WBC > 8000/µL	0.85	0.19	2.33	1.60	3.39	**<0.0001**

The table shows the results of the Cox regression analysis for mortality risk. The mean follow-up time was 2.3 years. Sex, age, BMI, and pack years were included as covariates. B indicates the unstandardized estimate, SE its standard error, HR the hazard ratio (=exp(B)), CI the confidence interval. When the analysis was repeated while including the BODE-Index and/or the intake of oral or inhaled corticosteroids, or Hb concentration or SaO_2,_ or heart failure or coronary artery disease as additional covariates, OxyHem and WBC remained significant predictors, whereby 14.1% of patients presented with OxyHem <12.5 g/dL and 41.6% with WBC > 8000/µL.

### Sensitivity analyses

The significant associations with OxyHem and WBC count were preserved when additionally including the BODE-Index as a predictor that was also linked to mortality. They were also maintained when including the intake of inhaled and oral corticosteroids as potential confounders, either alone or in addition to the BODE-Index. Additionally, the inclusion of either SaO_2_ or hemoglobin concentration as predictor did not affect their predictive significance. Moreover, the Cox regression models were confirmed by Bootstrap analysis based on 1000 samples each, and the numerical estimates and confidence intervals regarding OxyHem and WBC remained virtually the same. The linear correlation coefficient between CaO_2_ and OxyHem was 0.9999. Due to this extremely strong relationship, all significant findings regarding OxyHem also applied to CaO_2_, but in the analysis we targeted on OxyHem owing to its greater simplicity. The results regarding the mortality analysis were not essentially altered, when using a censored survival variable comprising more cases. When additionally introducing current smoking status as predictor, the HR for OxyHem became 2.98 and that for WBC 2.27, both of which were still significant. When additionally introducing oral or inhaled steroids as predictors, the HR for OxyHem remained at 2.77 and that for WBC at 2.20, both of which were still significant. The introduction of FEV_1_%pred as additional predictor did not affect the predictive value of OxyHem (see e-Table 7 in the supplement). Moreover, its value was only weakly affected (HR 2.22, p < 0.001) by the additional introduction of LTOT as predictor, the presence of which had a negative predictive value (HR 3.10, p < 0.001).

## Discussion

The present study had the aim to examine associations of blood gases (BG) with disease burden and prognosis in patients with stable COPD, focusing on the concentration of oxygenated hemoglobin (OxyHem) as a simple surrogate marker of oxygen content. To evaluate the magnitude of BG effects, we compared them with those of comorbidities and inflammatory markers. The predictive value of BG for symptoms, exacerbation history, BODE-Index and mortality was similar to that of some comorbidities. Regarding mortality, BG also showed a predictive value incremental to that of comorbidities and inflammation. Importantly, the novel predictor OxyHem dominated other blood gas parameters and conferred most of the BG information in stable COPD. It has the advantage over CaO_2_ that it can be computed from the easily available measures Hb and SaO_2_ without need for the assessment of PaO_2_.

COPD is known to be associated with multiple comorbidities contributing to the history for hospitalisations and mortality^26,27^. We found that symptom burden (mMRC) and the BODE-Index were associated with cardiovascular comorbidities, osteoporosis, gastrointestinal and mental disorders and hyperlipidemia, the latter in a beneficial direction, as the presence of hyperlipidemia was associated with lower BODE values, in line with previous observations^28^. Exacerbation history was mainly associated with hyperuricemia, gastrointestinal disorders, sleep apnea and concomitant asthma, also in accordance with the literature^29–31^. Among the inflammatory markers that included three cytokines, only WBC count turned out to be consistently related to all outcomes. This was also true when the intake of corticosteroids as potential confounders^32–34^ was taken into account. In the Eclipse cohort, the pattern of inflammatory markers, called “inflammome”, was found to be not necessarily a constant feature of COPD but associated with worse outcomes if persisting over 1 year^35^. COSYCONET could not provide longitudinal data for cytokines to analyze this but we assume that OxyHem, or equivalently CaO_2_, is indicative of the long-term status, whereas WBC counts might exhibit more fluctuations in response to relatively recent events. In accordance with this, the HR for mortality was slightly lower for WBC than for OxyHem. While it is known that cardiovascular and malignant diseases are associated with elevated WBC counts^36,37^, reflecting their role as outcome predictors in both COPD and non-COPD patients^35,38,39^, CaO_2_ has been less investigated. Tables 1 and 2 illustrate that the COPD population studied by us showed the typical characteristics of large COPD cohorts, thus it is unlikely that our findings were peculiar for COSYCONET.

According to our results, BG contained information that was independent from comorbidities or inflammatory markers, which was most obvious regarding mortality. OxyHem was the strongest predictor and associated with all outcome measures analyzed in this study. BE was linked to the BODE-Index, and there was a trend towards an increased mortality if metabolic compensation in terms of elevated BE occurred. BE has already been described as an independent predictor of survival in patients with severe COPD and hypercapnic respiratory failure^5^. Our findings extend this observation to a broader panel of patients including those with moderate to mild airflow obstruction. In the present analysis, BE was not directly linked to a significant degree to exacerbations or symptoms, but in a previous network analysis we found an indirect association mediated via lung function and other BG measures^7^.

The superiority of OxyHem in its association with measurements of disease burden and prognosis seems reasonable on the basis that CaO_2_ quantifies the amount of oxygen (mL per 100 mL blood volume) available for tissues, which is important for proper organ function^40^. Accordingly, in a recent study we identified CaO_2_ but not SaO_2_ or PaO_2_ as relevant for cognitive impairment in COPD^41^. Tissue oxygenation also depends on cardiac output. If cardiac output increases as a response to a reduction in oxygen transport capacity, this might exert additional stress to the cardiovascular system, which probably becomes relevant in patients with COPD and cardiovascular comorbidities.

The physiological responses to chronic hypoxemia include the development of polycythemia, which, noteworthy enough, is associated with an improved outcome in severe COPD^42^. Anemia, however, is more frequent in COPD occurring in 7.5–17% of patients^43,44^. The pathomechanisms underlying anemia probably involve many factors and include systemic inflammation^45^. Anemia is generally associated with a worse outcome in chronic disorders, especially COPD^46,47^, and its effects are reflected in increased dyspnoea, reduced exercise capacity^44^ and quality of life^48^, and increased mortality in patients on LTOT^49^.

Due to the low solubility of oxygen in blood, the variation in the oxygen content arising from PaO_2_ is negligible^50^, thus CaO_2_ is – up to an error of the order of 1% or less – determined by the product of the hemoglobin concentration and the fractional oxygen saturation. This can be directly seen in the coefficients for the computation of CaO_2_ (=1.34 × Hb × SaO_2_ + 0.0031 × PaO_2_)^23,51^. Our proposal to use the simple product termed “OxyHem” and to omit the Hüfner factor of 1.34, was motivated by the fact that OxyHem is not just an alternative number but allows for a direct, intuitive interpretation as concentration of oxygenated hemoglobin in g/dL.

OxyHem seemed to combine the information contained in hemoglobin concentration and oxygen saturation in an efficient manner. Its association with mortality did not disappear when these two indices were introduced as additional predictors. Remarkably, the increase in the BODE-score corresponding to a reduction by 2 g/dL in OxyHem was only slightly less than that corresponding to a diagnosis of cardiac disease. It could be evaluated in further studies, whether OxyHem can even be used as a simple point-of-care parameter indicating the individual disease burden. It might also be considered to investigate this measure as a therapeutic goal in interventions, for example the initiation and guidance of LTOT.

## Limitations

The analyses regarding symptom burden, exacerbation history and BODE-Index showed the limitations inherent to a retrospective, cross-sectional design, while the analysis of survival was prospective and longitudinal. The observation period for survival was 3 years, but it seems unlikely that the significant associations would disappear with longer observation time, in line with the supplemental analysis using censored follow-up. We also used all-cause mortality data for analysis. There is, however, no reason to assume that the relative contribution from cardiovascular mortality was different from that observed in other studies, as we found an association of cardiovascular comorbidities with mortality. Moreover, the panel of inflammatory markers was limited. This, however, might not be a major flaw, as our study focused on common markers and even the BG measures involve more organs than the lung, e.g. via base excess and hemoglobin.

The strength of our study is the large, high quality data set comprising a broad spectrum of COPD patients.

## Conclusions

In patients with stable COPD, the concentration of oxygenated hemoglobin (OxyHem), i.e. the product of hemoglobin concentration and fractional oxygen saturation, was most informative among blood gas measures and provided information on symptom burden, exacerbation history, BODE-Index and especially on mortality. This information added to that given by comorbidities and markers of inflammation. Values <12.5 g/dL predicted mortality with a HR of 2.77 independently from sex, age, BMI, pack years and cardiovascular comorbidities; for comparison, blood leukocytes >8000/µL showed a HR of 2.33. Thus, OxyHem, as a simple surrogate marker of the oxygen content CaO_2_, may be suitable as an additional measure for clinical use, especially for clinicians who lack equipment for the determination of PaO_2_.

## Supplementary information

Supplementary information.

## Data Availability

The basic data are part of the German COPD cohort COSYCONET (www.asconet.net/) and available upon request. There is a detailed procedure for this on the website of this network. Specifically, the data can be obtained by submission of a proposal which is evaluated by the steering committee.
