# Supplementary material for: Associations of oxygenated hemoglobin with disease burden and prognosis in stable COPD: Results from COSYCONET
Source: Sci Rep. 2020 Jun 29;10:10544. doi: 10.1038/s41598-020-67197-x (PMC7324620; doi:10.1038/s41598-020-67197-x)
Supplement: Supplementary file 1 — Supplementary information. [file 41598_2020_67197_MOESM1_ESM.docx]

**Associations of oxygenated hemoglobin with disease burden and prognosis in stable COPD: Results from COSYCONET**

^1^F.C. Trudzinski, ^2^R.A. Jörres, ^3^P. Alter, ^4^K. Kahnert, ^5,6^B. Waschki ^1^C. Herr, ^7^C. Kellerer, ^1^A. Omlor, ^3^C.F. Vogelmeier , ^8^S. Fähndrich, ^6^H. Watz, ^9^T. Welte, ^10^B. Jany, ^3^S. Söhler, ^11^F. Biertz, ^12^F. Herth, ^13^H.-U. Kauczor,^1^R. Bals, on behalf of the COSYCONET consortium

^1^Department of Internal Medicine V - Pulmonology, Allergology, Critical Care Care Medicine, Saarland University Hospital, Homburg, Germany.

^2^Institute and Outpatient Clinic for Occupational, Social and Environmental Medicine, Ludwig Maximilians University (LMU), Comprehensive Pneumology Center Munich (CPC-M), Member of the German Center for Lung Research (DZL), Munich, Germany.

^3^Department of Medicine, Pulmonary and Critical Care Medicine, Philipps University of Marburg (UMR), Member of the German Center for Lung Research (DZL), Marburg, Germany.

^4^Department of Internal Medicine V, University Hospital, LMU Munich, Comprehensive Pneumology Center, Member of the German Center for Lung Research (DZL), Munich, Germany.

^5^ Department of General and Interventional Cardiology, University Heart Center Hamburg, Hamburg, Germany

^6^Pulmonary Research Institute at LungenClinic Grosshansdorf, Airway Research Center North (ARCN), Member of the German Center for Lung Research (DZL), Grosshansdorf, Germany.

^7^TUM School of Medicine, Institute of General Practice and Health Services Research, Technical University of Munich, Orleansstraße 47, 81667, Munich, Germany.

^8^Department of Pneumology, University Hospital Freiburg, Freiburg, Germany.

^9^Clinic for Pneumology, Hannover Medical School, Biomedical Research in Endstage and Obstructive Lung Disease Hannover (BREATH), Member of the German Center for Lung Research, Hannover, Germany.

^10^Department of Internal Medicine, Medical Mission Hospital, Academic Teaching Hospital, Julius Maximilian University of Würzburg, Würzburg, Germany.

^11^Institute for Biostatistics, Hannover Medical School, Hannover, Germany.

^12^Department of Pneumology and Critical Care Medicine, Thoraxklinik University of Heidelberg, Translational Lung Research Center Heidelberg (TLRC-H), Member of the German Center for Lung Research (DZL), Heidelberg, Germany.

^13^Department of Diagnostic and Interventional Radiology, Heidelberg University Hospital, Translational Lung Research Center Heidelberg (TLRC-H) member of the German Center of Lung Research, Heidelberg, Germany.

**e-Appendix 1**

**The COSYCONET Study-Group comprises:**

Andreas, Stefan (Lungenfachklinik, Immenhausen); Bals, Robert Universitätsklinikum des Saarlandes); Behr, Jürgen and Kahnert, Kathrin (Klinikum der Ludwig-Maximilians-Universität München); Bewig, Burkhard (Universitätsklinikum Schleswig Holstein); Buhl, Roland (Universitätsmedizin der Johannes-Gutenberg-Universität Mainz); Ewert, Ralf and Stubbe, Beate (Universitätsmedizin Greifswald); Ficker, Joachim H. (Klinikum Nürnberg, Paracelsus Medizinische Privatuniversität Nürnberg); Gogol, Manfred (Institut für Gerontologie, Universität Heidelberg); Grohé, Christian (Ev. Lungenklinik Berlin); Hauck, Rainer (Kliniken Südostbayern AG, Kreisklinik Bad Reichenhall); Held, Matthias and Jany, Berthold (Klinikum Würzburg Mitte gGmbH, Standort Missioklinik); Henke, Markus (Asklepios Fachkliniken München-Gauting);Herth, Felix (Thoraxklinik Heidelberg gGmbH); Höffken, Gerd (Fachkrankenhaus Coswig GmbH); Katus, Hugo A. (Universitätsklinikum Heidelberg); Kirsten, Anne-Marie and Watz, Henrik (Pneumologisches Forschungsinstitut an der Lungenclinic Grosshansdorf GmbH); Koczulla, Rembert and Kenn, Klaus (Schön Klinik Berchtesgadener Land); Kronsbein, Juliane (Berufsgenossenschaftliches Universitätsklinikum Bergmannsheil, Bochum); Kropf-Sanchen, Cornelia (Universitätsklinikum Ulm); Lange, Christoph and Zabel, Peter (Forschungszentrum Borstel); Pfeifer, Michael (Klinik Donaustauf); Randerath, Winfried J. (Wissenschaftliches Institut Bethanien e. V., Solingen); Seeger, Werner (Justus-Liebig-Universität Gießen); Studnicka, Michael (Uniklinikum Salzburg); Taube, Christian and Teschler, Helmut (Ruhrlandklinik gGmbH Essen); Timmermann, Hartmut (Hamburger Institut für Therapieforschung GmbH); Virchow, J. Christian (Universitätsklinikum Rostock); Vogelmeier, Claus (Universitätsklinikum Gießen und Marburg GmbH, Standort Marburg); Wagner, Ulrich (Klinik Löwenstein gGmbH); Welte, Tobias (Medizinische Hochschule Hannover); Wirtz, Hubert (Universitätsklinikum Leipzig)

**e-Appendix 2**

**Determination of biomarkers**

IL-6, IL-8, and TNF-α were determined using a premixed Luminex high sensitivity cytokine magnetic bead assay (HSCYTMAG60SPMX13, EMD Millipore Corp., St. Charles, Missouri 63304, USA). Fibrinogen was assessed in citrate stabilized plasma samples using a premixed Luminex cytometric bead assay (HCVD3MAG-67K, EMD Millipore Corp., St. Charles, Missouri 63304, USA). All samples were measured on a MagPix device using xPonent 4.2 (EMD Millipore Corp., St. Charles, Missouri 63304, USA) for data acquisition and Milliplex Analyst (Vigenetech, EMD Millipore Corp., St. Charles, Missouri 63304, USA) for data analysis.

**e-Appendix 3**

**Blood gas analyzers available in the study centers:**

ABL 5, 90, 520, 550, 555, 700, 720, 800, 825 (Radiometer, Copenhagen, Denmark); OPTI blood gas analyzer (OPTI Medical Systems Inc., Roswell, Georgia, USA); RAPIDLab 1245 System, RAPIDPoint 400 and 405 (Siemens Healthcare GmbH, Erlangen, Germany); Omni S6 and Cobas b 221 (Roche Diagnostics, Mannheim, Germany); Chiron 248 (Chiron Diagnostics, Colchester, UK), GEM 3000, 3500, premier 4000 (Werfen, Barcelona, Spain); Nova (Nova Biomedical, Waltham MA, USA).

| **Variables** | **B** | **SE** | **HR** | **95%CI for HR** | | **P value** |
| --- | --- | --- | --- | --- | --- | --- |
|  |  |  |  | **Lower** | **Upper** |  |
| Sex (f vs m) | -0.60 | 0.23 | 0.55 | 0.35 | 0.87 | **0.0108** |
| Age (y) | 0.08 | 0.01 | 1.08 | 1.05 | 1.11 | **<0.0001** |
| BMI (kg/m^2^) | -0.05 | 0.02 | 0.95 | 0.91 | 0.99 | **0.0184** |
| Pack years | 0.00 | 0.00 | 1.00 | 0.99 | 1.00 | 0.6371 |
| Diabetes | -0.32 | 0.29 | 0.73 | 0.41 | 1.29 | 0.2753 |
| Hyperlipidemia | -0.25 | 0.20 | 0.78 | 0.53 | 1.15 | 0.2041 |
| Hyperuricemia | 0.22 | 0.22 | 1.25 | 0.81 | 1.92 | 0.3096 |
| Gastrointestinal | 0.34 | 0.19 | 1.40 | 0.97 | 2.03 | 0.0706 |
| Hypertension | 0.47 | 0.21 | 1.60 | 1.07 | 2.40 | **0.0235** |
| Cor. art. dis. | 0.30 | 0.23 | 1.35 | 0.87 | 2.11 | 0.1827 |
| Heart failure | 0.61 | 0.31 | 1.83 | 1.01 | 3.34 | **0.0480** |
| Osteoporosis | 0.41 | 0.24 | 1.51 | 0.95 | 2.41 | 0.0813 |
| Psychiatric | 0.49 | 0.22 | 1.63 | 1.07 | 2.48 | **0.0242** |
| Sleep apnea | -0.13 | 0.33 | 0.88 | 0.46 | 1.69 | 0.7033 |
| Asthma | -0.20 | 0.25 | 0.82 | 0.50 | 1.34 | 0.4227 |

**e-Table 1:** Cox proportional hazard regression analyses for mortality and comorbidities as predictors, with sex, age, BMI and pack years as covariates (see Figure 1). B indicates the unstandardized estimate, SE its standard error, HR the hazard ratio (=exp(B)), CI the confidence interval

| **Variables** | **B** | **SE** | **HR** | **95%CI for HR** | | **P value** |
| --- | --- | --- | --- | --- | --- | --- |
|  |  |  |  | **Lower** | **Upper** |  |
| Sex (f vs m) | -0.79 | 0.23 | 0.45 | 0.29 | 0.71 | **0.0007** |
| Age (y) | 0.06 | 0.01 | 1.06 | 1.04 | 1.09 | **<0.0001** |
| BMI (kg/m^2^) | -0.06 | 0.02 | 0.94 | 0.91 | 0.98 | **0.0067** |
| Pack years | 0.00 | 0.00 | 1.00 | 0.99 | 1.00 | 0.6584 |
| SaO_2_ (%) | -0.05 | 0.06 | 0.95 | 0.85 | 1.06 | 0.3765 |
| PaO_2_ (mmHg) | -0.01 | 0.02 | 0.99 | 0.95 | 1.03 | 0.5604 |
| PaCO_2_ (mmHg) | -0.04 | 0.05 | 0.96 | 0.87 | 1.07 | 0.4551 |
| pH | -5.72 | 7.26 | 0.00 | 0.00 | 4995.97 | 0.4309 |
| BE (mmol/L) | 0.19 | 0.10 | 1.21 | 0.99 | 1.46 | 0.0603 |
| OxyHem (g/dL) | -0.29 | 0.08 | 0.75 | 0.64 | 0.87 | **0.0002** |

**e-Table 2:** Cox proportional hazard regression analyses for mortality and the set of blood gas parameters as predictors, with sex, age, BMI and pack years as covariates (see Figure 1). B indicates the unstandardized estimate, SE its standard error, HR the hazard ratio (=exp(B)), CI the confidence interval

| **Tables** | **B** | **SE** | **HR** | **95%CI for HR** | | **P value** |
| --- | --- | --- | --- | --- | --- | --- |
|  |  |  |  | **Lower** | **Upper** |  |
| Sex (f vs m) | -0.59 | 0.23 | 0.56 | 0.35 | 0.88 | **0.0116** |
| Age (y) | 0.08 | 0.01 | 1.09 | 1.06 | 1.11 | **<0.0001** |
| BMI (kg/m^2^) | -0.05 | 0.02 | 0.95 | 0.91 | 0.99 | **0.0232** |
| Pack years | 0.00 | 0.00 | 1.00 | 1.00 | 1.01 | 0.6829 |
| WBC (1000 /µL) | 0.09 | 0.02 | 1.10 | 1.06 | 1.14 | **<0.0001** |
| CRP (mg/dL) | 0.02 | 0.03 | 1.02 | 0.96 | 1.08 | 0.4863 |
| Fibrinogen (g/L) | 0.12 | 0.06 | 1.12 | 0.99 | 1.27 | 0.0632 |
| IL-6 (pg/mL) | 0.00 | 0.00 | 1.00 | 1.00 | 1.00 | 0.5736 |
| IL-8 (pg/mL) | 0.00 | 0.00 | 1.00 | 1.00 | 1.00 | 0.2779 |
| TNF-α (pg/mL) | -0.01 | 0.01 | 0.99 | 0.98 | 1.01 | 0.3393 |

**e-Table 3:** Cox proportional hazard regression analyses for mortality and the set of inflammatory markers as predictors, with sex, age, BMI and pack years as covariates (see Figure 1). B indicates the unstandardized estimate, SE its standard error, HR the hazard ratio (=exp(B)), CI the confidence interval

**e-Figure 1**: Receiver operating characteristic (ROC) curves using comorbidities (blue line). blood gases (red line) and their combination (green line) versus the combination of comorbidities and inflammatory parameters (orange line) to predict symptoms **(A),** exacerbations **(B)** and BODE Index (cut-off value 2) **(C).** Sex, age, body mass index and pack years were kept as covariates in all analyses


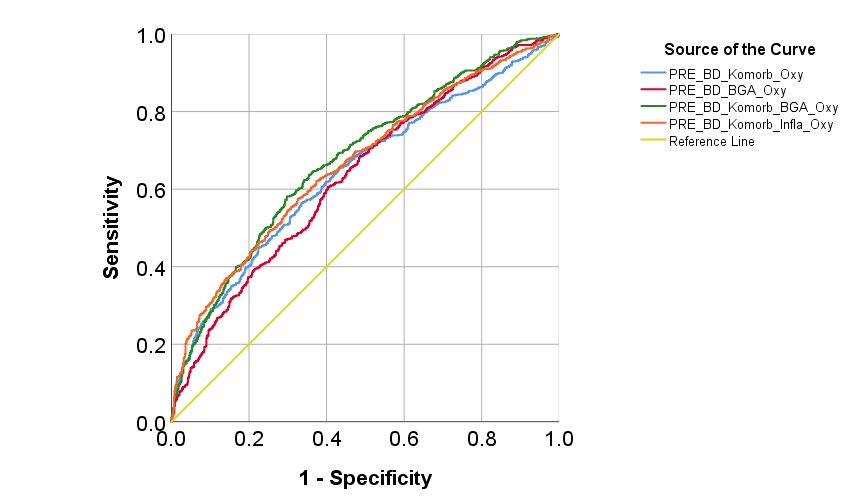

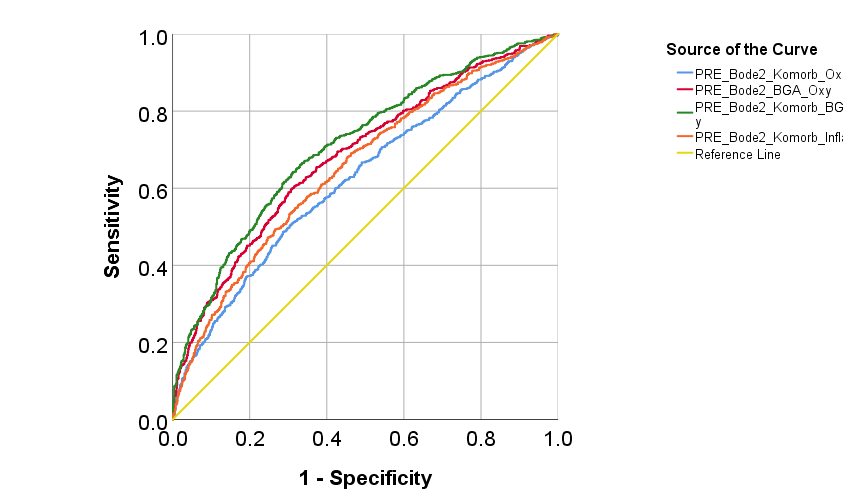

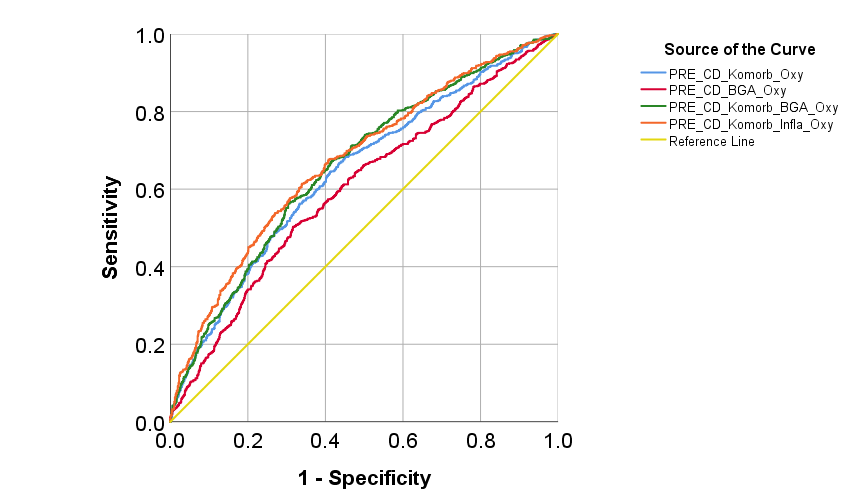


A

B

C

| **Test Result** | **AUC** | **SE**^a^ | **Asymptotic 95%CI** | | **Sign.^b^** |
| --- | --- | --- | --- | --- | --- |
|  |  |  | **Lower** | **Upper** |  |
| **Symptoms** |  |  |  |  |  |
| Comorbidities | 0.644 | 0.013 | 0.618 | 0.670 | **<0.0001** |
| Blood gases | 0.634 | 0.013 | 0.608 | 0.660 | **<0.0001** |
| Comorbidities & blood gases | 0.679 | 0.013 | 0.654 | 0.704 | **<0.0001** |
| Comorb. & inflammatory par. | 0.666 | 0.013 | 0.640 | 0.691 | **<0.0001** |
| **Exacerbation risk** |  |  |  |  |  |
| Comorbidities | 0.644 | 0.014 | 0.616 | 0.671 | **<0.0001** |
| Blood gases | 0.600 | 0.014 | 0.572 | 0.628 | **<0.0001** |
| Comorbidities & blood gases | 0.660 | 0.014 | 0.633 | 0.687 | **<0.0001** |
| Comorb. & inflammatory par. | 0.673 | 0.014 | 0.646 | 0.699 | **<0.0001** |
| **Bode Index (cut-off value 2)** |  |  |  |  |  |
| Comorbidities | 0.625 | 0.014 | 0.598 | 0.652 | **<0.0001** |
| Blood gases | 0.684 | 0.013 | 0.658 | 0.709 | **<0.0001** |
| Comorbidities & blood gases | 0.711 | 0.013 | 0.686 | 0.736 | **<0.0001** |
| Comorb. & inflammatory par. | 0.655 | 0.013 | 0.629 | 0.681 | **<0.0001** |

**e-Table 4:** Area under the ROC curve (AUC), its standard errors (SE) and 95% confidence intervals (see **e-Figure 1**). Comorbidities included diabetes, hyperlipidemia, hyperuricemia, gastrointestinal disorders, hypertension, coronary artery disease, heart failure, osteoporosis, psychiatric disorders, sleep apnea and asthma. Blood gas parameters were SaO_2_, PaO_2_, PaCO_2_, pH, BE and OxyHem. Inflammatory parameters comprised WBC, CRP, fibrinogen, IL-6, IL-8 and TNF-α. ^a^Under the nonparametric assumption, ^b^Null hypothesis: true area = 0.5

| **Variables** |  | |  | **95%CI** | | **p value** |
| --- | --- | --- | --- | --- | --- | --- |
|  | **B** | **SE** | **Beta** | **Lower Bound** | **Upper Bound** |  |
| Sex (f vs m) | -0.11 | 0.10 | -0.03 | -0.31 | 0.09 | 0.2684 |
| Age (y) | -0.01 | 0.01 | -0.04 | -0.02 | 0.00 | 0.1104 |
| BMI (kg/m^2^) | -0.03 | 0.01 | -0.08 | -0.05 | -0.01 | **0.0005** |
| Pack years | 0.00 | 0.00 | 0.00 | 0.00 | 0.00 | 0.8487 |
| Hyperlipidemia | -0.35 | 0.09 | -0.09 | -0.53 | -0.16 | **0.0002** |
| Osteoporosis | 0.75 | 0.13 | 0.13 | 0.50 | 1.00 | **<0.0001** |
| Heart failure | 0.53 | 0.20 | 0.06 | 0.14 | 0.92 | **0.0078** |
| Cor. art. disease | 0.43 | 0.13 | 0.08 | 0.19 | 0.68 | **0.0006** |
| WBC (1000 /µL) | 0.15 | 0.02 | 0.18 | 0.11 | 0.18 | **<0.0001** |
| OxyHem (g/dL) | -0.21 | 0.04 | -0.13 | -0.28 | -0.13 | **<0.0001** |

**e-Table 5**: Results of the linear regression analysis for the outcome variable BODE-Index. B indicates the unstandardized estimate, SE its standard error, Beta the standardized estimate, CI its confidence interval. The regression coefficients shown here provided the basis for the predicted changes shown in **Figure 2**.

| **Predictors** | **B** | **SE** | **HR** | **95%CI for HR** | | **P value** |
| --- | --- | --- | --- | --- | --- | --- |
|  |  |  |  | **Lower** | **Upper** |  |
| Sex (f vs m) | -0.56 | 0.23 | 0.57 | 0.36 | 0.89 | **0.0135** |
| Age (y) | 0.07 | 0.01 | 1.07 | 1.04 | 1.09 | **<0.0001** |
| BMI (kg/m^2^) | -0.06 | 0.02 | 0.94 | 0.90 | 0.98 | **0.0033** |
| Packyears | 0.00 | 0.00 | 1.00 | 0.99 | 1.00 | 0.6209 |
| Cor. artery disease | 0.29 | 0.22 | 1.33 | 0.87 | 2.05 | 0.1917 |
| Heart failure | 0.39 | 0.31 | 1.47 | 0.81 | 2.69 | 0.2088 |
| OxyHem <12.5 g/dL | 0.98 | 0.21 | 2.66 | 1.77 | 3.99 | **<0.0001** |
| WBC >8000/µL | 0.82 | 0.19 | 2.26 | 1.55 | 3.29 | **<0.0001** |

**e-Table 6:** Results of the Cox regression analysis for mortality risk with anthropometric covariates and heart failure and coronary artery disease as predictors, in addition to OxyHem and WBC. B indicates the standardized estimate, SE its standard error, HR the hazard ratio (=exp(B)), CI the confidence interval.

| **Predictor** | **B** | **SE** | **HR** | **95%CI for HR** | | **P value** |
| --- | --- | --- | --- | --- | --- | --- |
|  |  |  |  | **Lower** | **Upper** |  |
| Sex (f vs m) | -1.75 | 0.37 | 0.17 | 0.08 | 0.36 | <0.0001 |
| Age (y) | 0.03 | 0.02 | 1.03 | 1.00 | 1.06 | 0.090 |
| BMI (kg/m2) | -0.06 | 0.02 | 0.94 | 0.90 | 0.98 | 0.005 |
| Packyears | 0.00 | 0.00 | 1.00 | 0.99 | 1.00 | 0.685 |
| OxyHem <12.5 (g/dL) | 0.96 | 0.21 | 2.60 | 1.74 | 3.90 | <0.0001 |
| WBC >8000/µL | 0.80 | 0.19 | 2.22 | 1.52 | 3.23 | <0.0001 |
| FEV1 (%pred.) | -1.24 | 0.34 | 0.29 | 0.15 | 0.56 | <0.0001 |

**e-Table 7:** Results of the Cox regression analysis for mortality risk with anthropometric covariates OxyHem, WBC and FEV1(%pred). B indicates the standardized estimate, SE its standard error, HR the hazard ratio (=exp(B)), CI the confidence interval.
